# Prenatal diagnosis of infratentorial anomalies: ultrasound, fetal MRI, and implications for counseling

**DOI:** 10.1007/s00247-025-06272-9

**Published:** 2025-06-16

**Authors:** Teresa Chapman, David M. Mirsky, J. Igor Iruretagoyena, Carolina V. Guimaraes

**Affiliations:** 1 Department of Radiology, University of Wisconsin–Madison, 600 Highland Ave, Madison, WI 53792 USA; 2 Department of Radiology, University of Colorado Anschutz Medical Campus, Aurora, USA; 3 Department of Obstetrics and Gynecology, University of Wisconsin–Madison, Madison, USA; 4 Department of Radiology, University of North Carolina at Chapel Hill, Chapel Hill, USA

**Keywords:** Brain, Posterior fossa, Cerebellar anomalies, Congenital, Prenatal counseling

## Abstract

Assessment of the fetal brain is a routine part of standard and detailed prenatal ultrasound. Further evaluation of central nervous system abnormalities identified by prenatal ultrasound is a common indication for fetal MRI. Infratentorial malformations identified by diagnostic imaging may be isolated or may occur with supratentorial anomalies and with multisystemic anomalies or syndromes. The interpretation of posterior fossa abnormalities and their implications for diagnosis and prenatal counseling can benefit from recognizing the dominant morphological feature. This review discusses the ultrasound and MRI characteristics of developmental anomalies affecting the cerebellum and brainstem, focusing on their primary imaging characteristic: cystic anomalies, cerebellar dysgenesis, and brainstem malformations. Key findings on imaging are provided to guide the radiologist in arriving at a reasonable diagnosis. Genetic implications are also provided.

**Graphical Abstract:**

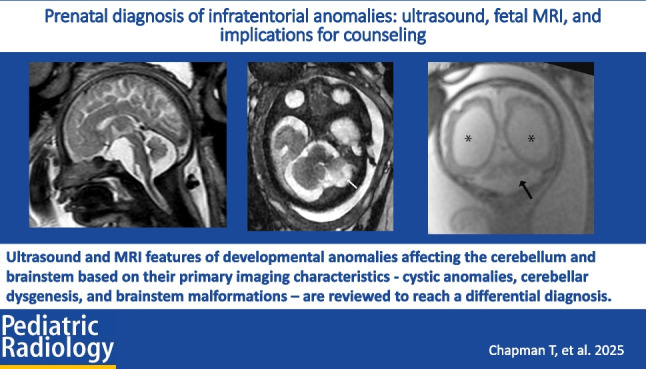

## Introduction

Prenatal identification of infratentorial malformations by ultrasound (US) and magnetic resonance imaging (MRI) is essential for antenatal care, aiding both parental counseling and planning neonatal management. Infants and children with cerebellar and brainstem malformations may experience various postnatal clinical challenges, exhibiting a broad spectrum of severity. The potential effects of infratentorial anomalies include motor difficulties, brainstem and cranial nerve dysfunction, seizures, and a range of neurocognitive deficits [1]. Identifying the type of posterior fossa malformation offers insights into postnatal outcomes and may assist in detecting associated anomalies, both within and outside the central nervous system.

Because the cerebellum’s development is protracted and continues into the early postnatal period [2, 3], cerebellar anomalies may be missed or undetectable during fetal development. Additionally, recognizing morphologic abnormalities of the brainstem and cerebellum can be challenging due to limitations in prenatal imaging resolution [4, 5]. Further complicating diagnosis in this area are confusing changes in terminology and descriptions of different genetic and developmental entities [1, 6, 7].

Posterior fossa malformations may occur either in isolation or in association with supratentorial anomalies, highlighting the importance of thoroughly inspecting the entire fetal brain [2, 6, 8, 9]. Infratentorial anomalies can be categorized based on the dominant feature, as follows: posterior fossa cystic anomalies, cerebellar dysgenesis, and brainstem malformations. While these features may co-occur, examining the fetal anatomy to classify the findings appropriately may assist in further investigating the brain morphology and provide an optimal diagnosis or reasonable differential of etiologies to guide counseling. This review utilizes the aforementioned categorization to illustrate the US and MRI characteristics of various developmental anomalies affecting the cerebellum and brainstem using the above categorization.

## Prenatal ultrasound and MRI of posterior fossa

**Fig. 1:**
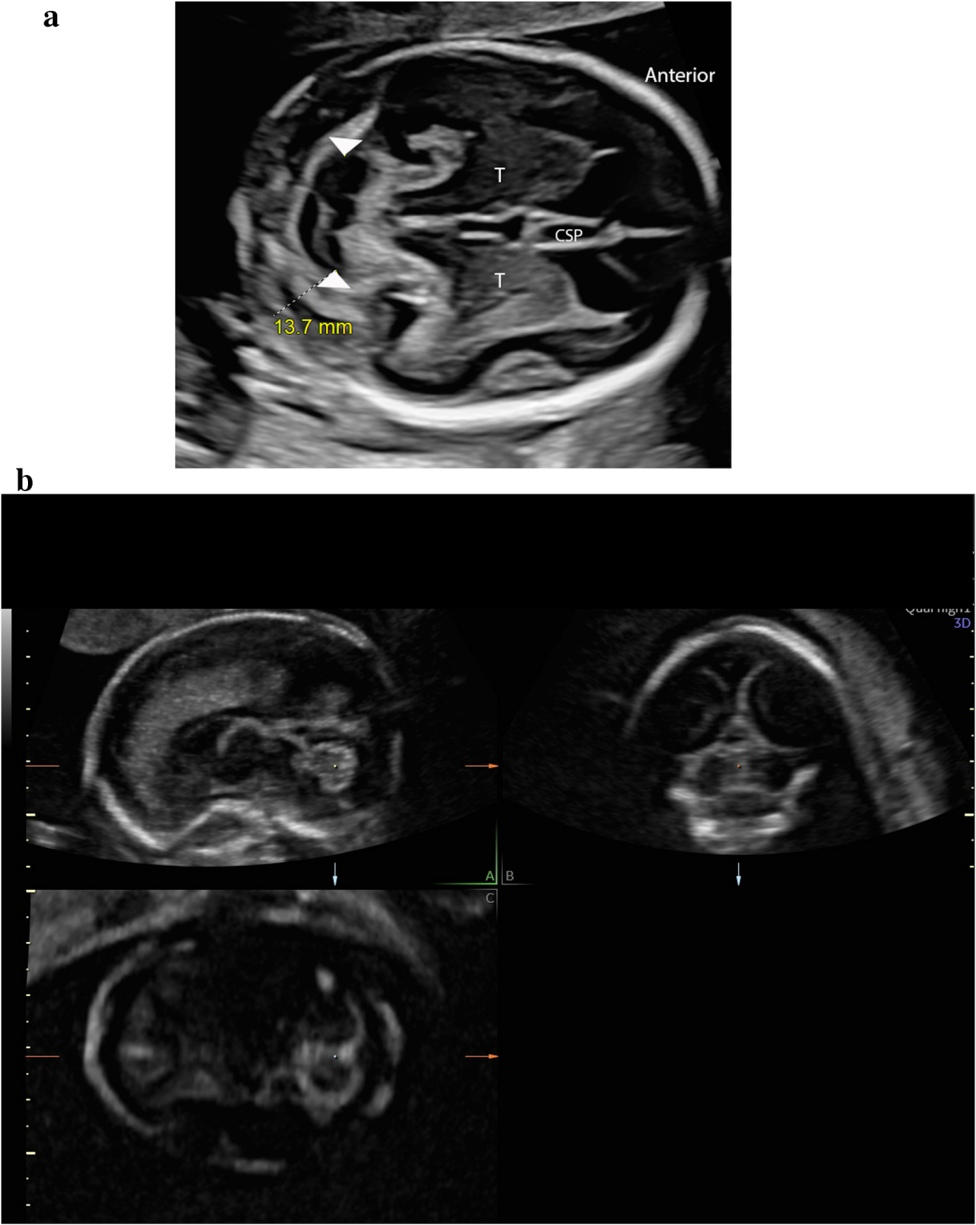
Ultrasound assessment of the posterior fossa in a fetus at 19 4/7 weeks. **a** Suboccipitobregmatic transverse view of the fetal head aligns the lower cavum septum pellucidum (CSP), thalamic nuclei (T), and cerebellum for accurate assessment of the transverse cerebellar diameter (TCD). TCD measures 13.7 mm (*between arrowheads*), which is less than the 1st percentile (50th percentile for TCD is 19.7 mm at this GA)^10^. **b** Three-dimensional ultrasound allows for the reconstruction of posterior fossa imaging to provide sagittal assessment of the vermis and craniocaudal measurement to improve the accuracy of diagnosis (*dot* denotes center of vermis in all three planes)

A comprehensive elaboration of normal fetal brain imaging has been provided in this Special Edition. Briefly, an assessment of the posterior fossa solid tissue includes the morphology, size, and echogenicity of the brainstem and cerebellum on ultrasound, along with the size, shape, and signal properties on MRI. Standard second-trimester fetal brain assessment of the posterior fossa includes images of the cerebellar hemispheres and cisterna magna, with biometry of the transverse cerebellar diameter (TCD) in the axial plane and comparing that to published normative values for gestational age (Fig. 1a) [10, 11]. Detailed second-trimester ultrasound includes evaluation of the posterior fossa in both axial and sagittal planes, evaluating the TCD, vermis, fourth ventricle, and cisterna magna [12]. Visualization of the vermis in the mid-sagittal plane is beneficial, although acquiring this view by ultrasound may be technically challenging depending on fetal positioning or maternal body habitus. Three-dimensional (3D) ultrasound may assist in these situations, enabling visualization by acquiring data in the transverse plane and reconstructing images of the brainstem and vermis in the sagittal plane (Fig. 1b). 3D imaging also allows the operator to select the appropriate plane for measurements, the optimal slice thickness, and the ideal filters that best delineate the parenchymal details [13].

Concerns for brain or spine anomalies identified by ultrasound can be safely evaluated with fetal MR imaging [14]. Multiplanar fast T2-weighted MR images provide excellent anatomical details of the vermis and the cerebellar hemispheres, complementing prenatal ultrasound [15]. Additional T1 and echo planar MR sequences may be helpful in detecting hemorrhage. Fetal brain biometry is available for second- and third-trimester fetuses [16–20].

## Cystic anomalies of the posterior fossa

Infratentorial cystic developmental anomalies are characterized by a prominent or thin-walled cerebrospinal fluid (CSF) collection, with or without enlargement of the posterior fossa size [21]. Interpretation of cystic posterior fossa anomalies must be made in conjunction with an assessment of the cerebellum and brainstem, including size, morphology, and echogenicity or signal. The conditions discussed in this section include mega cisterna magna, Blake pouch cyst, Dandy-Walker malformation, and arachnoid cysts.

### Mega cisterna magna

**Fig. 2:**
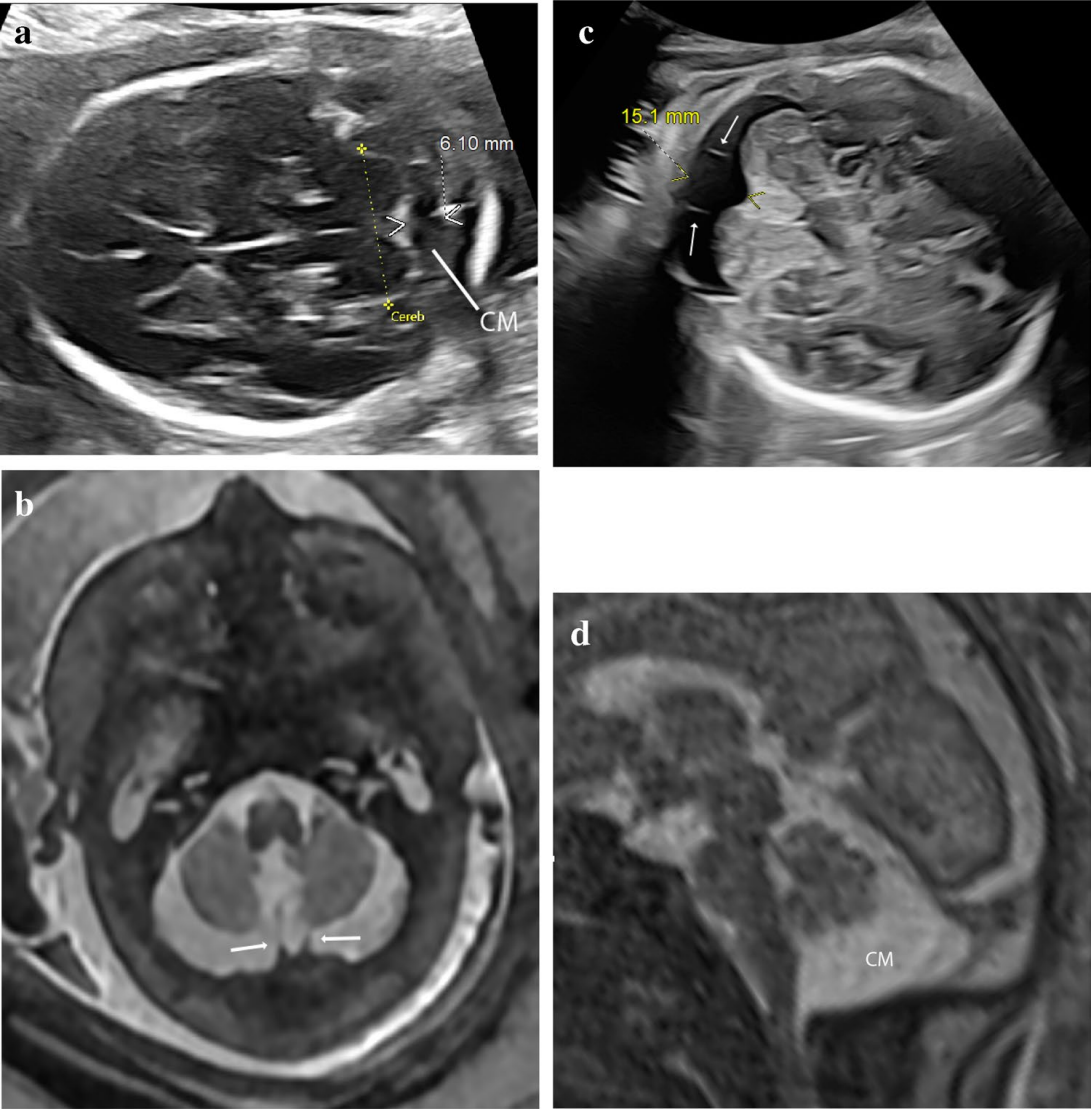
Normal cisterna magna and mega cisterna magna. **a** Suboccipitobregmatic transverse view of the fetal head at 22 0/7 weeks demonstrates TCD (*between calipers*) and AP measurement of the cisterna magna (*between arrowheads*), normal at 6.1 mm. **b** Axial T2 single-shot fast-spin echo (SSFSE) through the lower fetal posterior fossa at 24 2/7 weeks shows the typical appearance of the falx cerebelli (*arrows*). **c**-**d** Mega cisterna magna in a fetus at 30 0/7 weeks gestation. Transverse ultrasound (**c**) shows a widened cisterna magna measuring greater than 10 mm in AP dimension. Note linear echogenicities (*arrows*) of the falx cerebelli. Duplication of the falx cerebelli without deviation is supportive of mega cisterna magna. Fetal brain MRI sagittal SSFSE image (**d**) of the midline posterior fossa at the same gestational age demonstrates abnormal uniform prominence of the cisterna magna (CM). No mass effect or cyst wall was evident, and the parenchyma was normal

The cisterna magna is a CSF-containing space located posterior to the cerebellum. It appears anechoic on ultrasound and follows CSF-intensity on all MR sequences. As measured between the inferior margin of the vermis and the posterior rim of the foramen magnum, the normal cisterna magna is less than 10 mm in the anterior-posterior (AP) dimension in the axial plane (Fig. 2a). The falx cerebelli, which are thin pachymeningeal reflections, may be observed in the paramedian location of the cisterna magna, extending from the inner surface of the occipital bone anteriorly toward the junction of the cerebellar hemispheres and vermis [22]. These structures are visible in the axial and coronal planes as either thin linear echogenic structures on ultrasound or T2-dark linear structures on MRI (Fig. 2b) and may assume various shapes, such as branching (Y-shaped) or curvilinear (U-shaped) [23].

One entity to consider when confronted with an enlarged retrocerebellar fluid space is a mega cisterna magna, defined as a widened CSF space dorsal to the cerebellum, with an AP dimension greater than 10 mm (Fig. 2c). There is free communication between a normally formed fourth ventricle and the subarachnoid space through a patent median foramen, along with an absence of any cyst in the posterior fossa. This is a diagnosis of exclusion. Importantly, the cerebellar parenchyma, biometry, and ventricles should be normal, and there should be no mass effect on the cerebellar tissues to imply the presence of an arachnoid cyst. The T2-weighted signal of the fluid space should match that of the subarachnoid spaces, without alteration to suggest a lack of free communication. Notably, the falx cerebelli is non-displaced and has been observed to be multiplicated in the setting of mega cisterna magna [23] (Fig. 2d).

### Persistent Blake pouch cyst

A persistent Blake pouch cyst (BPC) is characterized by the upward rotation of the vermis away from the brainstem, accompanied by an increased width of the tegmento-vermian angle due to a space-occupying, thin-walled cyst [24, 25]. The conventional definition of this disorder includes normal formation of the vermis and cerebellar hemispheres; however, minimal mass effect on the nodulus and uvula (the inferior ventral-most lobules of the vermis) may occur and could theoretically impact the development of the inferior vermis. A persistent BPC is essentially a manifestation of arrested development. Normally, there is a transient evagination of the membranous fourth ventricular roof as an ependyma-lined diverticulum extending posteriorly into the primitive meninx caudal to the cerebellum. The walls of this diverticulum should fenestrate to create the foramen of Magendie, allowing communication with the subarachnoid space, occurring variably between the 7th week and the 4th month of gestation [7, 25]. The normal absorption of the ependymal lining and involution of the pouch leads to incorporation of the CSF space as the arachnoid-lined cisterna magna [25]. If this membrane fails to fenestrate, it will distend with CSF and form a space-occupying cyst. Depending on the patency of the foramina, a persistent BPC may lead to hydrocephalus, which is a crucial determinant of prognosis.

**Fig. 3:**
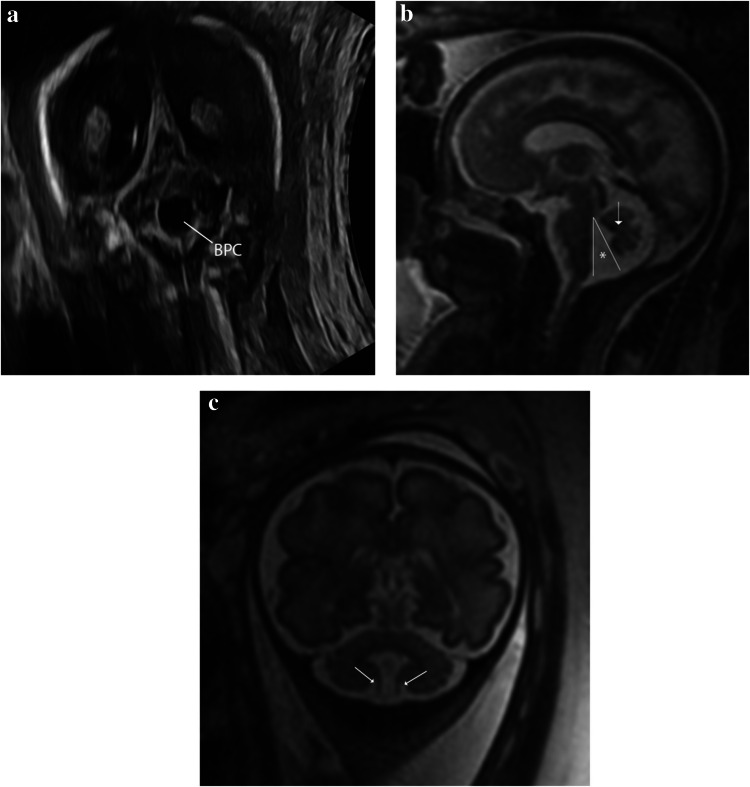
Persistent Blake pouch cyst versus vermian hypoplasia. **a** Prenatal ultrasound of the fetal head in the coronal plane at 20 6/7 weeks shows an anechoic space between the two cerebellar hemispheres, reflecting the CSF-filled Blake pouch cyst (BPC). **b**, **c** A different case of suspected BPC versus mild vermian hypoplasia is shown by fetal MRI at 30 3/7 weeks, with upward rotation of the vermis and enlarged tegmento-vermian angle (*asterisk*) evident on the midline sagittal SSFSE view (**b**). Note also the sharp fastigial recess (*arrow*), helping to distinguish BPC from a Dandy-Walker phenotype. Although a normal shape of the vermis is maintained, the primary fissure is prominent, and the subjective volume of the inferior vermis is slightly less than expected, warranting consideration of inferior vermian hypoplasia. However, postnatal brain MRI at term showed the normal appearance of the vermis and a normal, narrow tegmento-vermian angle (not shown). Coronal SSFSE (**c**) shows the medial position of the taenia-tela choroidea complex (*white arrows*), as seen in the presence of BPC

The diagnosis of this condition using ultrasound or MRI relies on midline sagittal imaging to confirm the normal appearance of the vermis, lateral displacement of the cisterna magna septa, upward rotation of the vermis, and an increased tegmento-vermian angle, giving the appearance of a midline cyst at the caudal aspect of the vermis (Fig. 3) [25]. The position of the fourth ventricle choroid plexus is also helpful in this diagnosis; it is ectopic, elevated along the undersurface of the vermis when a Blake pouch cyst persists. However, because the choroid plexus is challenging to visualize, the taenia–tela choroidea complex (TTCC), a small chain of structures (taenia – tela choroidea – choroid), may be easier to observe than the choroid plexus of the fourth ventricle [26]. It can sometimes be detected on fetal and postnatal MRI. If the tela choroidea is positioned superiorly and medially (“up and in”), this suggests a persistent BPC, as opposed to malpositioning of the TTCC inferiorly and laterally (“down and out”), which supports the diagnosis of a Dandy-Walker phenotype, discussed below [26].

Larger BPCs can present a diagnostic challenge when they compress the inferior vermis and expand the fastigial recess of the fourth ventricle. In this case, inferior vermian hypoplasia and the Dandy-Walker phenotype should also be considered. Additional diagnoses to consider include inferior vermian dysgenesis with ex-vacuo prominence of the cisterna magna and inferior vermian compression by a retrocerebellar arachnoid cyst. It is also important to note that BPC may co-exist with inferior vermian hypoplasia. Surveillance by prenatal ultrasound to monitor for the development of hydrocephalus and the enlargement of the cystic space is appropriate. In instances that are not diagnostically clear, repeating fetal MRI later in gestation may help clarify the etiology, and a postnatal brain MRI may be necessary to re-evaluate the vermis. Persistent BPC has been found to spontaneously resolve in over 50% of cases between 24 weeks and 26 weeks of gestation due to delayed fenestration of the fourth ventricle [24].

### Dandy-Walker malformation

**Fig. 4:**
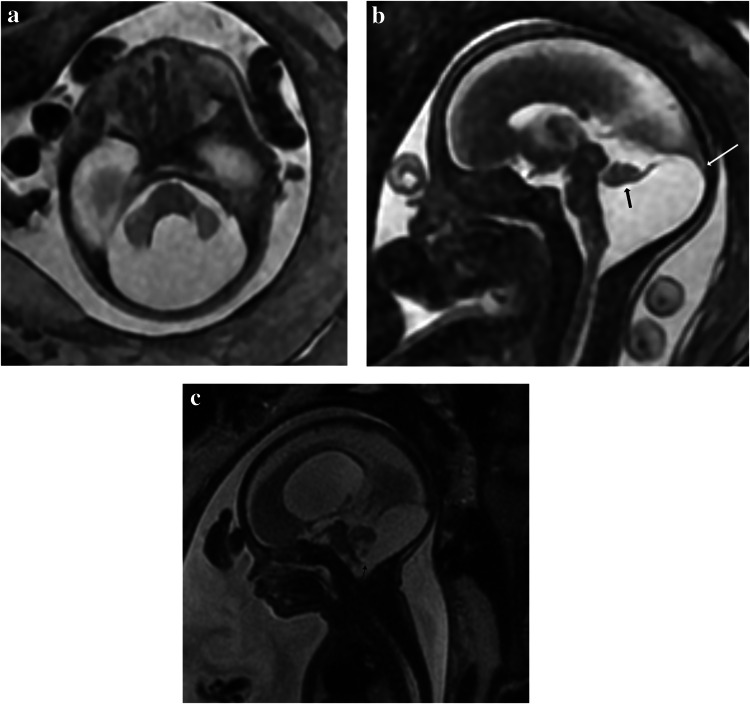
Dandy-Walker malformation (DWM). Fetal brain MRI was performed at 23 0/7 weeks. Axial T2 SSFSE (**a**) demonstrates pronounced enlargement of the posterior fossa and abnormal CSF space between the cerebellar hemispheres in the axial plane through the mid posterior fossa in place of the expected vermis. On the sagittal T2 fast steady-state image through the midline posterior fossa (**b**), an abnormally elevated torcula (*white arrow*), small vermis, enlargement of the fourth ventricle with a blunted fastigium (*black arrow*), and communication with the retrocerebellar CSF space are apparent, diagnostic of DWM. **c** A different case of DWM in a fetus at 21 1/7 weeks, with a sagittal T2 SSFSE off-midline sagittal projection demonstrating the tela choroidea (*black arrow*) positioned ectopically, low and lateral, typical of the Dandy-Walker phenotype, in contrast to high and medial positioning with BPC

The classic Dandy-Walker malformation (DWM) was originally described as a severe hindbrain malformation involving the congenital absence of the foramina of Magendie and Luschka [27, 28], characterized by the following anatomic features: (1) an abnormally large posterior fossa (high position of the tentorium); (2) hypoplasia or aplasia of the vermis; (3) upward rotation of the vermis; and (4) a dilated fourth ventricle with the absence of its normal fastigial point and open communication between the ventricle and the retrocerebellar CSF space (Fig. 4a, b). Recent literature on the embryology and imaging manifestations of this entity does not rely on the presence of fourth ventricular outflow obstruction or enlargement of the posterior fossa to make the diagnosis of a Dandy-Walker phenotype [3, 26, 29]. When the TTCC is visualized and is malpositioned inferiorly and laterally (“down and out”), this indicates a DWM (Fig. 4c) [26]. Hydrocephalus may be present but is not necessary for the diagnosis.

Due to the variability in diagnostic criteria and terminology over recent decades, many experts agree that a detailed description of the posterior fossa abnormalities and associated anomalies is crucial for accurate prognosis. Regardless of the absolute percentage of DWM cases with associated anomalies, it is widely accepted that a significant percentage of prenatal and postnatal cases of DWM have additional malformations involving the central nervous system (CNS) and non-CNS systems [3, 30, 31]. Associated brain anomalies that have been described include hydrocephalus, agenesis of the corpus callosum, holoprosencephaly, encephalocele, cortical malformations, and gray matter heterotopias. The most commonly reported anomalies outside the central nervous system include congenital heart disease (seen in approximately 25%), polycystic kidney disease, and cleft lip palate [30, 31]. Therefore, a careful assessment of the fetal brain, spine, face, and body at the time of imaging is critical upon recognition of a Dandy-Walker phenotype.

### Arachnoid cyst

**Fig. 5:**
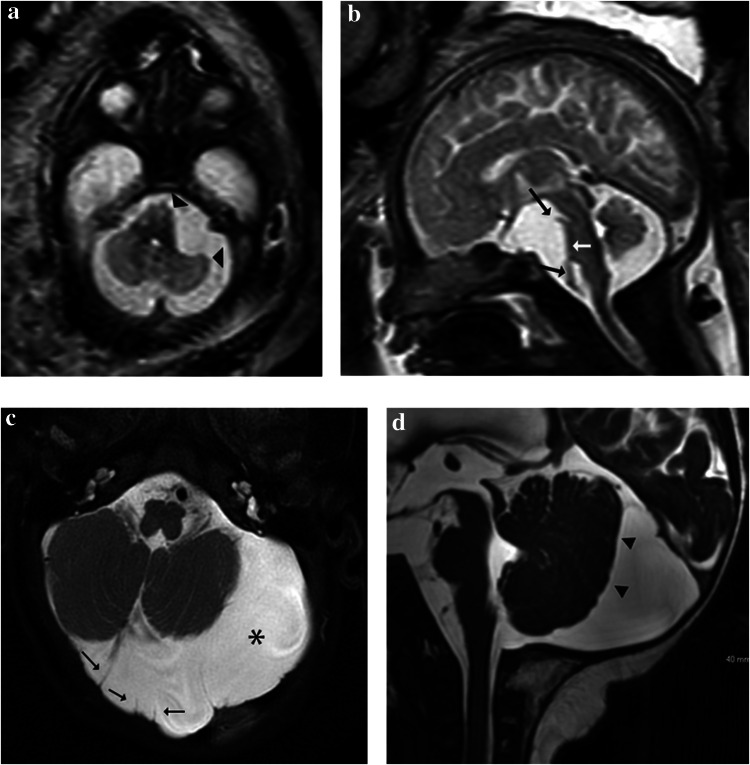
Arachnoid cysts. Left cerebellopontine angle arachnoid cyst diagnosed by fetal MRI (**a**) at 27 4/7 weeks on axial T2 SSFSE based on mass effect upon the left pons and anterior cerebellar hemisphere (*arrowheads*). Prepontine arachnoid cyst diagnosed by fetal MRI (**b**) at 33 4/7 weeks on sagittal T2 SSFSE based on mass effect upon the ventral pons (*white arrow*) and dorsal displacement of the basilar artery flow void (*black arrows* in **b**). Based on its location between the chiasmatic, interpeduncular, and prepontine cisterns, this represents a “membrane of Lilliquist” arachnoid cyst. **c**, **d** Postnatal brain T2-weighted MRI of a different patient at age 6 months with posterior fossa arachnoid cyst. Axial (**c**) and sagittal (**d**) images demonstrate mass effect upon the left cerebellum and upon the vermis posteriorly (*arrowheads* in **d**) with unilateral expansion of the left posterior fossa fluid space (*asterisk*). Normal falx cerebelli is present in the inferior midline cisterna magna (*arrows* in **c**)

Arachnoid cysts are sacs formed by the duplication or splitting of the arachnoid membrane and contain CSF-like fluid [32]. The walls of these cysts are imperceptible on imaging, and their presence can be inferred by mass effect (Fig. 5). The main risks associated with an arachnoid cyst are enlargement and obstructive hydrocephalus, although most remain stable in size. The majority of arachnoid cysts are supratentorial [32–35]. Due to the confined space, arachnoid cysts in the posterior fossa may have neurodevelopmental consequences, caused by mass effect upon adjacent brain structures, although many have no deleterious outcome. Serial US is necessary to monitor both the cyst size and the size of the ventricles, and to exclude any additional anomalies. Postnatal complications of arachnoid cysts include macrocephaly and obstructive hydrocephalus, which may require neurosurgical intervention such as intracranial shunts, craniotomy, and cyst fenestration [36]. The prenatal estimation of outcomes will depend on location, size, and impact on adjacent parenchyma, growth trajectory, and associated anomalies [32, 33].

### Outcomes of cystic posterior fossa anomalies

While BPC and mega cisterna magna have a statistically much more favorable prognosis than DWM and are less frequently associated with anomalies, the prognosis of DWM remains complex. The clinical features and developmental outcomes of DWM vary widely, ranging from intellectual disability to autism or may remain undiagnosed until discovered incidentally in adulthood [1]. A thorough fetal anatomical assessment is critical, since abnormal development is more common in cases with associated anomalies. Approximately 90% to 95% of fetuses with either BPC or mega cisterna magna and no associated anomalies have normal neurologic development, compared to only 50% of those with DWM [1, 37–39].

## Cerebellar hypoplasia

Cerebellar hypoplasia refers to the underdevelopment of the cerebellum. The diagnosis of cerebellar hypoplasia is distinct from the previously described infratentorial cystic anomalies; while the posterior fossa CSF spaces may appear vacuous and empty, the tentorium and the TTCC are normally positioned. Cerebellar hypoplasia is characteristic of many different disorders, including genetic and acquired conditions. The clinical prognosis is variable and reflects the underlying cause, which will be elaborated upon here.

### Isolated inferior vermian hypoplasia

**Fig. 6:**
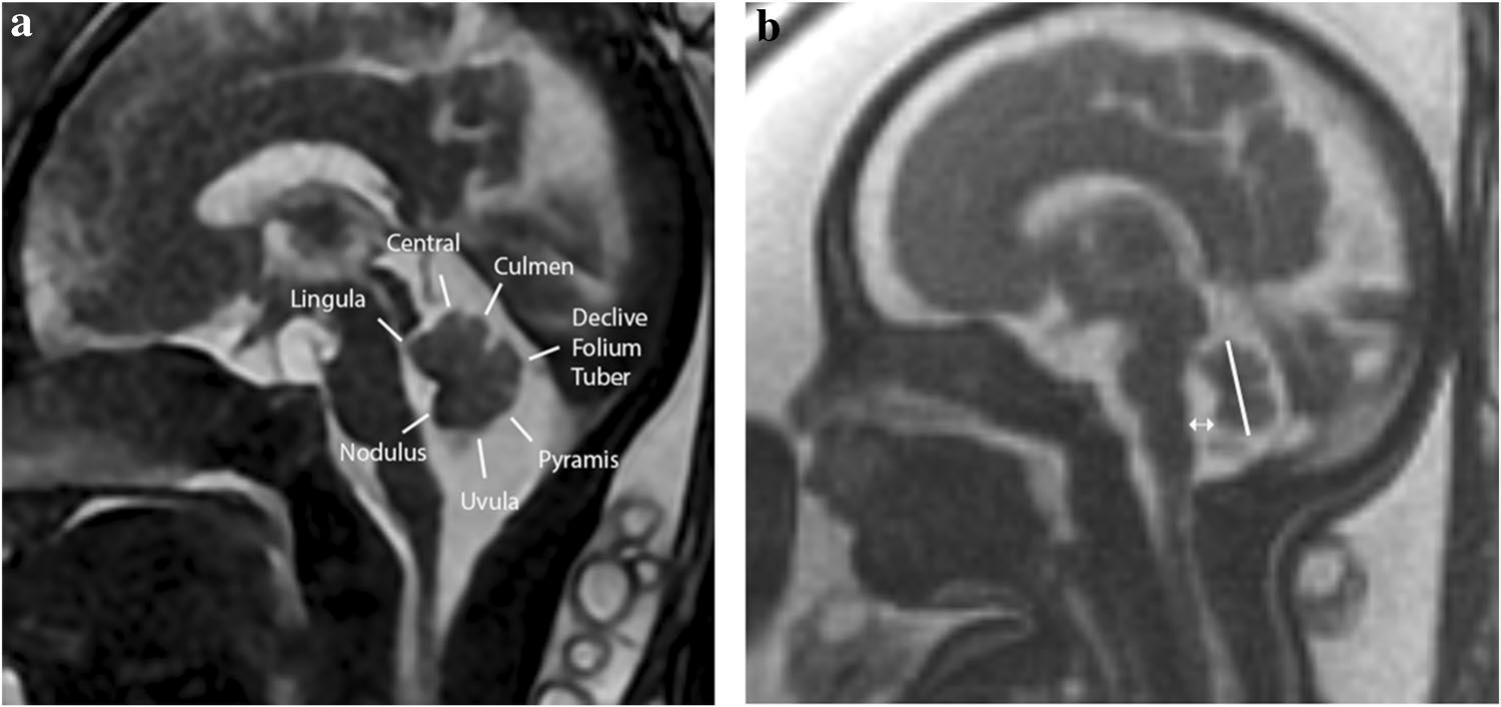
Vermian anatomy and isolated inferior vermian hypoplasia. **a** Fetal MRI performed on a 1.5-T scanner at 25 2/7 weeks delineates seven of nine cerebellar vermian lobules. **b** Fetal MRI midline sagittal T2 fast steady-state image performed at 31 1/7 weeks shows mild inferior vermian hypoplasia with the vermis craniocaudal dimension slightly smaller than expected for gestational age (*white line*). Inferior vermian distance is minimally widened, measuring 4 mm (*arrows*). Additionally, note the relatively equal amount of vermian tissue present above and below the fastigium-declive axis, whereas normally, the inferior vermis is slightly larger

The vermis is the midline lobe of the cerebellum and contributes to motor functions, vestibulo-ocular coordination, and cognition [40]. Nine distinct lobules of the vermis develop separately from the cerebellar hemispheres. Different parts of the vermis arise from different sections of the mesial primordium, leading to segmental vermian abnormalities [7]. Seven of the nine vermian lobules can be identified in a normal fetus from week 22 of gestation onward using 1.5-tesla MRI [41] (Fig. 6a). An anatomical assessment of vermian development by either ultrasound or MRI relies on a midline sagittal view of the posterior fossa and includes visualization of the following features in a normal vermis after week 18 of gestation: (1) complete coverage of the fourth ventricle with a sharply defined fastigial point; (2) a narrow tegmento-vermian angle that is typically close to zero and always less than 12° if under 24 months GA or less than 8° if over 24 months GA (an alternative measurement is the inferior vermian distance, defined as the anteroposterior distance from the inferior-most vermis to the brainstem, normally less than 4 mm); (3) visualization of the primary fissure; (4) normal measurements of the craniocaudal height and AP dimension of the vermis; and (5) normal echogenicity and/or signal properties of the vermis [15, 20, 21, 42, 43]. The diagnosis of isolated inferior vermian hypoplasia (IIVH) is established both qualitatively and quantitatively. The inferior-ventral portion of the vermis appears subjectively partially absent, and the height of the vermis is less than expected for gestational age, resulting in an abnormally widened tegmento-vermian angle and inferior vermian distance (Fig. 6b) [7, 43, 44]. Additionally, although not yet validated with outcomes studies, shared experience supports assessing the ratio of vermian tissue above and below a reference line from the fastigial point to the top of the declive (normally roughly 2:3) on a midline sagittal view. With IIVH, this ratio becomes abnormal after approximately 20 weeks of gestation, such that the inferior vermian area is equal to or less than the area of vermian tissue above the fastigium-declive line (Fig. 6b). Also of note, when significant vermian tilt is present in association with evidence of inferior vermian hypoplasia, a persistent Blake pouch cyst likely coexists.

The diagnosis of IIVH is made only if the fetal anatomy is otherwise normal and if no chromosomal anomaly is detected through routine prenatal laboratory studies. Because prenatal imaging is not sensitive enough to reliably detect all pathology that may be present in the developing brain, it is common practice to acquire postnatal brain MR imaging to evaluate subtle intracranial anomalies that were either not previously seen or were not yet apparent [45]. Research shows that children with postnatally confirmed IIVH exhibit favorable neurodevelopmental function at school age [46]. However, during the prenatal period, caution is warranted in both diagnosing IIVH and predicting the prognosis. One study [44] involving 19 cases of prenatally suspected isolated inferior vermian hypoplasia found that six patients had normal postnatal brain MRI. The remaining 13 patients with confirmed IIVH had overall good outcomes, with only mild developmental delays in a subset of infants. All infants were free of significant neurodevelopmental impairment and disability. Notably, overall developmental, behavioral, and functional scores tended to be lower among infants with IIVH compared to those with normal postnatal MRI studies [44].

### Hypoplasia of cerebellar hemispheres

Recognition of small cerebellar hemispheres warrants an examination of several potential etiologies, including infectious, genetic, toxic/metabolic, and genetic factors, as summarized in Table 1 [47]. When both the brainstem and cerebellum are reduced in size, a diagnosis of pontocerebellar hypoplasia should be considered, which will be discussed later.

**Table 1:** Etiologies known to be associated with cerebellar hypoplasia [1, 47]

Group of diseases	Etiologies to consider
Prenatal infection	Congenital cytomegalovirus infection
Prenatal teratogens	Antiepileptic drugs (phenytoin, valproic acid); retinoic acid; alcohol; cocaine
Chromosomal anomalies	Trisomy 13, 18, 21; partial trisomy 12q, monosomy 21a; trisomy 17 mosaicism; monosomy 1p36; de novo X; 8 translocation; 13q12.3-q14.11 deletion
Metabolic disorders	Adenylosuccinase deficiency; Smith-Lemli-Opitz syndrome; molybdenum cofactor deficiency and isolated sulfite oxidase deficiency; copper metabolism disease (SLC33 A1 mutation); Zellweger syndrome; nonketotic hyperglycinemia; mitochondrial disorders (Leigh disease, pyruvate dehydrogenase deficiency); mucopolysaccharisdoses types I and II
Genetic syndromes	Ritscher-Schinzel (3 C) syndrome; Hoyeraal-Hreidarsson syndrome; CHARGE syndrome; Endosteal sclerosis; oculocerebrocutaneous (Delleman) syndrome; microcephaly with simplified gyral pattern, epilepsy and permanent neonatal diabetes syndrome (IER3IP1 mutation); NF type 1; pseudo-TORCH syndrome; velocardiofacial syndrome; oculodentodigital syndrome; Cohen syndrome; Cri-du-chat (cat’s cry) syndrome; Pallister-Killian syndrome; Galloway-Mowat syndrome; Sengers syndrome; X-linked intellectual deficit-cerebellar hypoplasia.

*CHARGE*, coloboma, heart defects, atresia choanae (also known as choanal atresia), growth retardation, genital abnormalities, and ear abnormalities; *IER3IP1*, immediate early response 3 interacting protein 1; *NF*, neurofibromatosis; *SLC33 A1*, solute carrier family 33 member 1; *TORCH*, toxoplasmosis, others (syphilis, hepatitis B), rubella, cytomegalovirus, herpes simplex

**Fig. 7:**
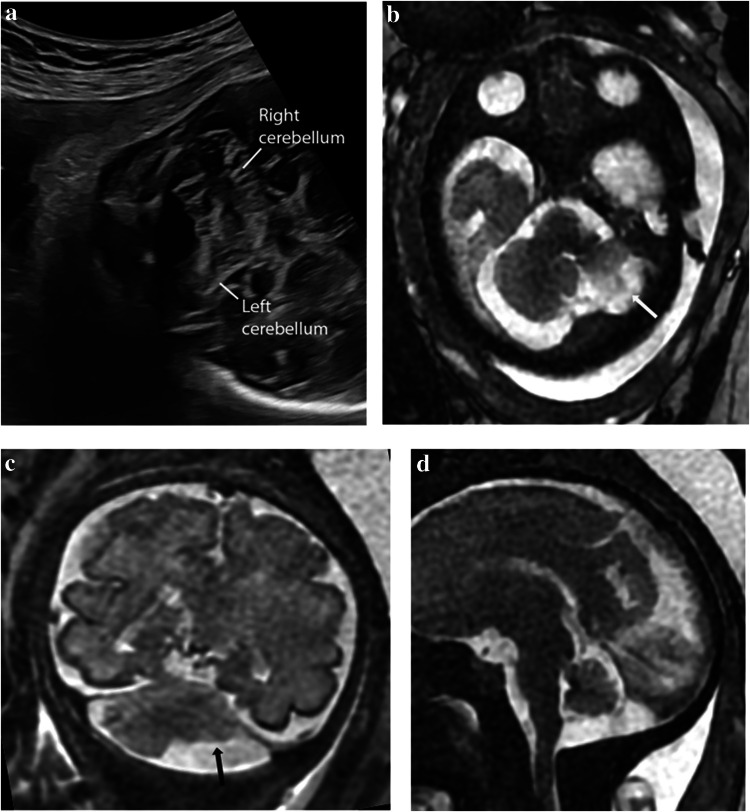
Unilateral cerebellar hypoplasia. Prenatal ultrasound (**a**) at 31 1/7 weeks shows underdevelopment of the left cerebellar hemisphere with loss of normal architecture. Same-day fetal brain MRI axial (**b**) and coronal (**c**) T2 single-shot fast-spin echo (SSFSE) images show a hypoplastic left cerebellar hemisphere (*arrows*). Midline sagittal T2 fast steady-state image (**d**) demonstrates a normal vermis. Etiology was favored to be an isolated injury. No blood products were appreciable on the heme-sensitive sequence (*not shown*)

Unilateral cerebellar hypoplasia is diagnosed when there is asymmetry in the size of the cerebellar hemispheres, with or without abnormal echogenicity or signal on the affected side of the cerebellum (Fig. 7). The vermis, the size of the posterior fossa, and the size of the fourth ventricle are otherwise normal. Most cases result from ischemic and/or hemorrhagic injury due to trauma, infection, vascular defects, blood dyscrasias, drug ingestion, and alloimmune or isoimmune thrombocytopenia. Less common conditions to consider include PHACE syndrome (facial hemangioma associated with anomalies of the posterior fossa, cerebral arteries, as well as cardiovascular and ocular alterations) [48] and familial porencephaly caused by COL4A1 and COL4A2 mutations [49].

The prognosis for most of these non-genetic cases of unilateral cerebellar hypoplasia is more favorable than that seen in the more complex scenario of bilateral cerebellar hypoplasia. Importantly, unilateral cerebellar hypoplasia with a normal vermis often results in a *normal* outcome [47]. Vermian involvement may indicate an increased risk of intellectual disability and autism [47].

### Cerebellar hypoplasia and lissencephaly

**Fig. 8:**
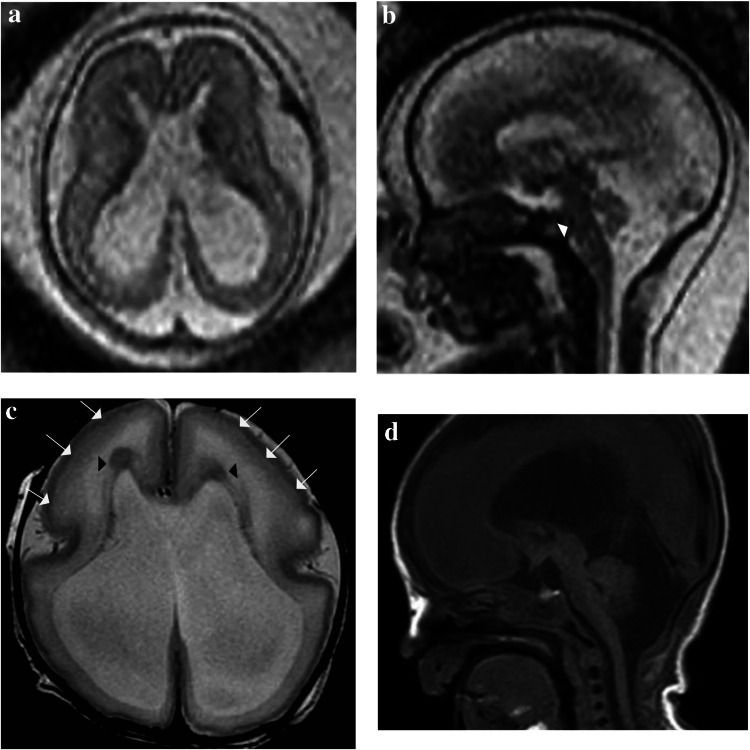
Vermian hypoplasia, brainstem hypoplasia, and lissencephaly. Fetal MRI axial supratentorial T2 single-shot fast-spin echo (**a**) performed at 22 weeks shows ventriculomegaly and a slightly shallow appearance of Sylvian fissures for age. Sagittal midline image (**b**) shows a small vermis and a flattened pons (*white arrowhead*). Postnatal MRI performed at 5 days of age shows lissencephaly. Axial T2 (**c**) sequence through the supratentorial brain shows severe ventriculomegaly, absent cerebral sulci aside from Sylvian fissures, an abnormal band of T2-isointense heterotopia in the bifrontal subcortical white matter (*white arrows*), and bifrontal periventricular heterotopic tissue (*black arrowheads*). Sagittal T1 (**d**) sequence through the midline brain redemonstrates the pontine and mild vermian hypoplasia seen on fetal MRI

Lissencephaly refers to a rare group of brain disorders in which the surface of the brain is abnormally smooth due to impaired neuronal migration during organogenesis [1, 50, 51]. The lissencephaly spectrum of brain malformations should be considered when observing vermian hypoplasia and/or bilateral cerebellar hypoplasia. Prenatal imaging may provide clues to the presence of cortical malformation, including the following abnormalities: abnormally shallow Sylvian fissures, delayed or premature appearance of expected sulci with gestation, an abnormally thick or thin cortex, an overtly simplified cortex, irregular ventricular borders, or intraparenchymal echogenic nodules (Fig. 8) [50, 52].

The lissencephaly spectrum of brain malformations may be caused by defects in the genes for reelin, its receptor, or tubulin. While the brain imaging findings for lissencephaly caused by any of these genetic defects may overlap, certain features typify each [1]. Reelin (*RELN*) regulates neuronal migration within both the cerebral cortex and cerebellum. Mutations of *RELN* with a recessive inheritance are known to cause a distinctive lissencephaly characterized by severe cerebellar hypoplasia with minimal foliation, referred to as lissencephaly with cerebellar hypoplasia (LCH) [53]. A lesser severity of cerebellar hypoplasia with some preservation of foliation may be caused by mutations in the *VLDLR* gene [1, 54], which encodes the very-low-density lipoprotein receptor and plays a role in suppressing neuronal invasion into the marginal zone of the developing neocortex [55]. In contrast to the brain MRI findings seen with *RELN* mutations, the degree of sulcal simplification, cortical thickening, and pachygyria is milder in *VLDLR*-associated lissencephaly [56].

**Fig. 9:**
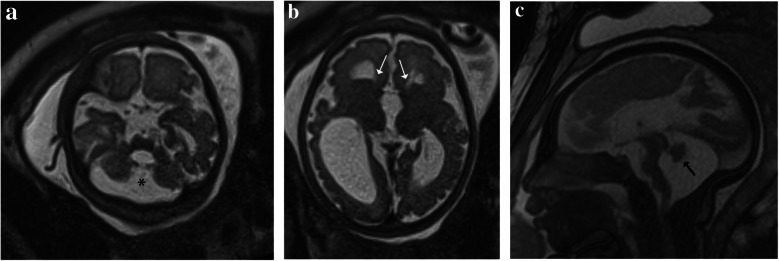
Tubulinopathy (*TUBB3* mutation) in a 31 6/7 week fetus. Fetal MRI axial T2 single-shot fast-spin echo (SSFSE) image of the posterior fossa (**a**) shows the absence of vermian tissue between the two cerebellar hemispheres (*asterisk*) and a diminutive brainstem, as well as an abnormal sulcation pattern in the visualized temporal lobes. SSFSE of the supratentorial fetal brain (**b**) shows asymmetric ventriculomegaly, abnormal sulcation, abnormal contours, and lack of defined architecture of the basal ganglia, with associated abnormally angular contours of the frontal horns medially (*white arrows*). Sagittal midline T2 fast steady-state image (**c**) shows marked vermian hypoplasia (*black arrow*), abnormally exaggerated pontine and cervical flexures of the brainstem giving a kinked appearance, and the absence of the corpus callosum

During brain development, alpha-tubulin partners with beta-tubulin to form microtubules that organize neuronal migration. Defects in the alpha- and beta-tubulin genes (including *TUBA1A*, *TUBA8*, *TUBB2A*, *TUBB2B*, *TUBB3*, *TUBB4A*, and *TUBB*) cause various intracranial anomalies, such as vermian hypoplasia, cerebellar dysgenesis (ranging from normal to severe), and brainstem hypoplasia, as well as supratentorial developmental anomalies like cortical dysgenesis (for example, agyria, pachygyria, dysgyria, subcortical band heterotopia, and polymicrogyria), corpus callosal dysgenesis, and basal ganglia dysplasia [57, 58]. Indicators on prenatal imaging, whether by ultrasound or MRI, that suggest a tubulinopathy in the setting of cerebellar hypoplasia include the following: callosal anomalies, ventricular asymmetry, dysmorphic and/or dilated frontal horns, abnormal sulcation, flattened pons, anteroposterior diameter of the pons less than the 5th centile, and brainstem asymmetry [59] (Fig. 9). Identifying dysplastic basal ganglia with absent internal capsule anterior limbs should be attempted, but may be challenging on prenatal imaging.

## Cerebellar dysgenesis

Cerebellar dysgenesis may inherently imply an abnormally small cerebellum, but we use this categorization primarily to emphasize a feature of abnormal morphology. There is a wide range of morphological abnormalities, as the etiologies leading to cerebellar dysgenesis vary significantly. The diagnoses discussed in this category include rhombencephalosynapsis and cerebellar cystic dysmorphism, as well as ⍺-dystroglycanopathy and other types of cobblestone malformations.

### Rhombencephalosynapsis

Rhombencephalosynapsis (RES) is a rare midline anomaly of the cerebellum, characterized by a partial to complete absence of the vermis, along with varying degrees of union of the cerebellar hemispheres and dentate nuclei [60, 61]. This condition has been linked to Gomez–Lopez-Hernandez (GLH) syndrome (RES plus scalp alopecia, abnormal head shape, often turricephaly, and low-set and/or posteriorly rotated ears) [61, 62] and the VACTERL-H association (vertebral anomalies, anorectal malformation, cardiac defects, tracheoesophageal fistula, renal anomalies, and limb abnormalities, along with hydrocephalus) [60, 61]. Beyond those associations, RES has also been reported with additional intracranial anomalies (heterotopias, cortical malformations, union of the colliculi, aqueductal stenosis, agenesis of the corpus callosum, flattening of the pons in AP dimension, lobar holoprosencephaly, and neural tube defects) [60, 61]. Even in the absence of a diagnosis of GLH syndrome, affected individuals may have an abnormal head shape, midface hypoplasia, low-set and/or posteriorly rotated ears, and hypertelorism [60].

**Fig. 10:**
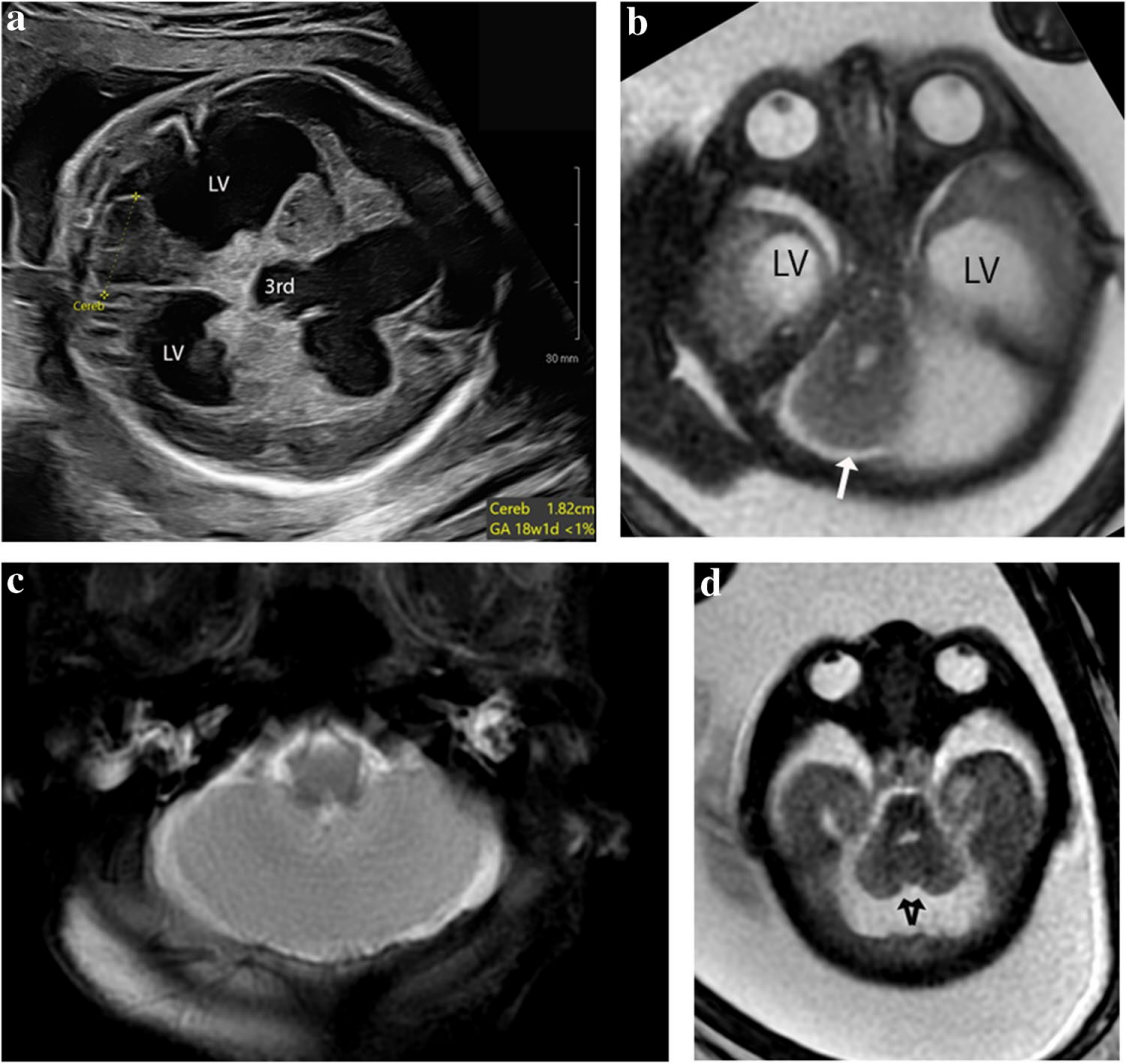
Rhombencephalosynapsis (RES). Prenatal ultrasound (**a**) at 26 3/7 weeks shows a transverse cerebellar diameter less than the first percentile for gestation. Severe supratentorial ventriculomegaly is also appreciable (*LV*, lateral ventricle; *3rd*, third ventricle). Subsequent fetal MRI performed at 32 2/7 weeks shows findings of RES. Axial T2 through the posterior fossa (**b**) demonstrates an abnormal contour of the dorsal cerebellum, with a continuous convex contour (*arrow*) and obvious severe supratentorial ventriculomegaly secondary to aqueductal stenosis. Postnatal brain MRI (**c**) axial T2 image through the posterior fossa shows the abnormal union of the two cerebellar hemispheres. **d** Normal appearance of the cerebellar vermis and hemispheres in the axial plane in a different fetus imaged at 31 weeks. Note normal clefts (*arrows*) between the vermis and hemispheres to compare to that seen with RES (**b**)

Prenatal diagnosis of RES is challenging. This diagnosis should be suspected when the following observations are made: (1) a small transverse cerebellar diameter below the 3rd percentile, (2) partial or complete absence of the vermis between the cerebellar hemispheres, creating a round-shaped cerebellum dorsally in the axial plane, (3) loss of the fastigial point with a trapezoidal or elongated shape of the fourth ventricle in the sagittal plane, and (4) hydrocephalus (Fig. 10a) [5]. As mentioned above, additional supratentorial and brainstem abnormalities may be present with RES; therefore, a careful inspection of the brain parenchyma for further findings is essential. Postnatal MRI reveals continuity of the folia and white matter across the midline without a distinct transition between the vermis and cerebellar hemispheres (Fig. 10b). Additionally, a reduced size of the posterior fossa has been observed in postnatal cases of RES with complete vermian absence [60]. Long-term cognitive outcomes vary from normal intelligence in isolated RES to severe impairment. Neurodevelopmental outcomes for RES closely correlate with the severity of midline union and associated anomalies, including obstructive hydrocephalus and the need for postnatal shunting [60].

### Cerebellar cystic dysmorphism

Cerebellar cysts are rare findings in neonates and children and are characteristic of alpha-dystroglycanopathies (including Walker-Warburg syndrome, muscle-eye-brain disease, and Fukuyama muscular dystrophy), but are much less commonly seen in GPR56-related encephalopathy, an autosomal recessive form of cobblestone-like frontoparietal polymicrogyria [63–65]. The term “cobblestone” is used to describe these entities due to the characteristic appearance of the cortical malformation on macroscopic examination, wherein defects in the pial basement membrane prevent proper attachment of the radial glial fibers, leading to the abnormal migration of neurons across the pial basement membrane into the subarachnoid space [65, 66]. Genetic defects associated with this group of disorders are listed in Table 2.

**Table 2:** Reported pathogenic variants responsible for alpha-dystroglycanopathies and other cobblestone malformations [1, 63, 73, 97, 98]

Diagnosis	Pathogenic variants
Walker-Warburg, muscle-eye-brain disease, Fukuyama muscular dystrophy	POMT1, POMT2, POMGnT1, FKTN, FKRP, LARGE, ISPD, B3GALNT2, DPM2, DPM3, GTDC2, B3GNT1, B3GNT2, SGK196, GMPPB, CGDC2, TMEM5,GTDC2, PGAP2
GPR56-related brain malformations	GPR56
Poretti-Boltshauser syndrome	LAMA1 defect

*B3GNT1*, *B3GNT2*, beta-1,3-N-acetylglucosaminyltransferase; *CGDC2*, CUB domain-containing protein 2; *DPM2,3*, dolichyl-phosphate mannosyltransferase subunit 2 and 3; *FKRP*, fukutin-related protein; *FKTN*, fukutin; *GMPPB*, GDP-mannose pyrophosphorylase; *GPR56*, adhesion G protein-coupled receptor G1; *ISPD*, isoprenoid synthase domain; *LAMA1*, alpha 1 subunit of laminin; *LARGE*, LARGE xylosyl- and glucuronyl transferase; *POMT1*, *POMT2*, *POMGnT1*, protein O-mannosyltransferase (POMT) enzyme complexes

The observation of cerebellar cysts should prompt consideration of alpha-dystroglycanopathies, a heterogeneous group of congenital muscular dystrophies (CMD). This spectrum of diseases arises from recessive mutations in multiple genes that lead to overlapping phenotypes. At the severe end of the spectrum is Walker-Warburg disease, characterized by small and dysplastic cerebellar hemispheres, a small vermis, a thinned and kinked brainstem, pontine cleft, dysplastic midbrain, hydrocephalus due to aqueductal stenosis, cobblestone cerebral cortex, abnormal white matter, and corpus callosal dysgenesis [63, 66–69]. In the middle of the spectrum is muscle-eye-brain disease, with neuroimaging features that include milder dysmorphism of the cerebellar hemispheres, milder brainstem hypoplasia, cerebral cortical malformations, and abnormal white matter [1, 63, 70]. Finally, the mildest manifestations are observed in Fukuyama muscular dystrophy, characterized by milder hypoplasia and dysmorphism of the cerebellar hemispheres, milder brainstem hypoplasia, and cortical malformations [1, 63, 67, 70].

**Fig. 11:**
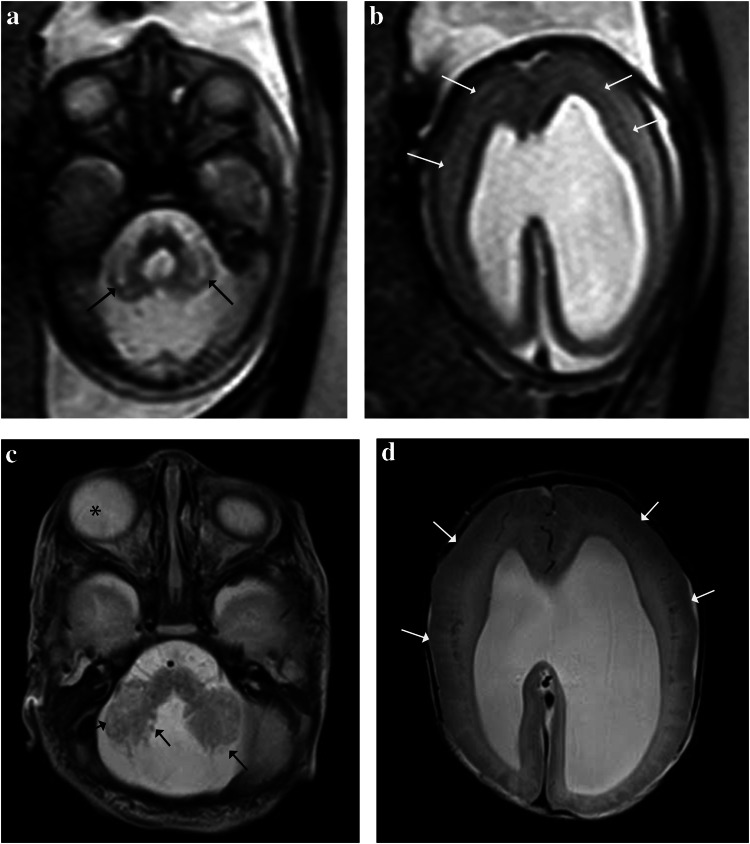
Cobblestone malformation in Walker-Warburg phenotype. Fetal MRI performed at 25 2/7 weeks shows features strongly suggestive of an alpha-dystroglycanopathy. Axial T2 SSFSE (**a**) through the posterior fossa shows small size of the cerebellum and subcortical hyperintense signal suggestive of cystic changes (*arrows*). Supratentorial axial SSFSE (**b**) demonstrates severe ventriculomegaly, simplified gyration for gestational age, and abnormal cerebral layering subcortical signal suggesting diffuse neuronal migrational abnormality. Postnatal brain MRI axial T2 through the posterior fossa at 1 week of age (**c**) shows severe dysmorphism and cystic changes of the cerebellar hemispheres (*arrows*). Buphthalmos (*asterisk*) is partially visualized on the right. Supratentorial axial T2 (**d**) shows ventriculomegaly and cobblestone malformation of the cerebral cortex (*arrows*)

Prenatal diagnosis is feasible by identifying various abnormalities summarized above. Infratentorial findings to investigate include cerebellar hypoplasia, peripheral cerebellar cystic changes, or signal alterations secondary to small peripheral cysts, brainstem hypoplasia, and brainstem kinking [71, 72]. Supratentorial findings that further indicate these genetic disorders include ventriculomegaly, abnormal sulcation, and gyral patterns on fetal MRI (resembling lissencephaly), as well as eye abnormalities (Fig. 11) [65, 71–74]. An additional prenatal sonographic finding associated with cobblestone malformations is a peripheral band of echogenicity surrounding the supratentorial structures [51]. However, identifying cobblestone malformations can be exceedingly challenging, even by fetal MRI.

## Brainstem malformations

Many of the infratentorial anomalies described above refer to brainstem malformations, particularly hypoplasia, which appears on imaging as flattening of the pons, a narrowed AP dimension of the pons, or distortion of the expected craniocaudal length ratio of the midbrain, pons, and medulla. Since many complex brain anomalies originate early in embryology, brainstem anatomy is often malformed [75]. In this final section, we will expand on the diagnoses of pontocerebellar hypoplasia, Joubert syndrome, and the implications of a kinked brainstem.

### Pontocerebellar hypoplasia

The diagnosis of pontocerebellar hypoplasia (PCH) is a distinct neurodegenerative disorder characterized by fetal onset and an autosomal recessive inheritance pattern. This condition results in developmental defects and progressive atrophy of the brainstem, along with hypoplasia or absence of the vermis, and cerebellar hemisphere hypoplasia [76–78]. Global developmental delay, which is typically severe, is a clinical hallmark of pontocerebellar hypoplasia [76, 77]. Additionally, feeding problems, seizures, microcephaly, hypotonia or hyperreflexia in infancy, and the later development of spasticity and involuntary movements during childhood may occur with some types [1].

**Fig. 12:**
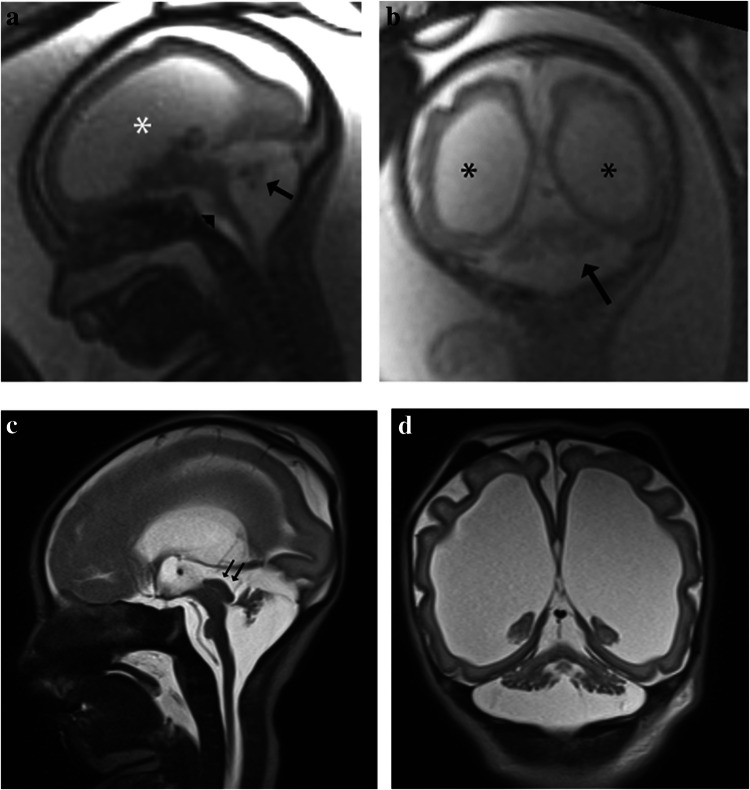
Pontocerebellar hypoplasia (type 4). Fetal MRI was performed at 32 1/7 weeks following ultrasound (*not shown*) documenting transverse cerebellar diameter <5th percentile, small vermis, and severe ventriculomegaly with cerebral mantle thinning. Sagittal (**a**) T2 fast steady-state image through the midline shows supratentorial ventriculomegaly (*asterisk*), severe flattening of the pons (*arrowhead*), and markedly small vermis (*arrow*). The absence of the corpus callosum (CC) versus pronounced thinning was unclear. Coronal (**b**) image through the posterior head shows the degree of parenchymal thinning and cerebellar hypoplasia. Postnatal brain MRI at 2 days of age, sagittal (**c**) and coronal (**d**) SSFSE images show the same findings as seen prenatally, with confirmation of absent CC and an additional note of tectal plate thickening (*double arrows*)

With the advances in genetic technologies, novel PCH genes continue to be identified. The two originally described types of PCH by Barth [77] have since expanded to nineteen types [76, 79]. In addition to the pontine and cerebellar hypoplasia observed, prenatal brain findings of PCH may include mild enlargement of the posterior fossa, thickening of the tectum, thinning or absence of the corpus callosum, and supratentorial ventriculomegaly [1] (Fig. 12). Notably, in some cases, the neurodegenerative process may not manifest with brain volume loss until the postnatal period and therefore may not be diagnosed prenatally.

In addition to the unique autosomal recessive diagnosis of PCH characterized by its fetal onset and progressive neurodegenerative features, the morphological combination of hypoplasia of the pons and cerebellum may be attributed to various other inherited and acquired disorders, as summarized in Table 3. Pontine tegmental cap dysplasia (PTCD) is a distinct, severe brainstem malformation to consider when confronted with severe flattening of the pons. PTCD manifests as an abnormal focal convexity of dysplastic tissue on the dorsal surface of the pons that protrudes into the fourth ventricle. This diagnosis also has a poor prognosis, resulting in severe motor and cognitive disorders as well as cranial nerve impairment [80, 81].

**Table 3:** Etiologies associated with pontine and cerebellar hypoplasia

Group of diseases	Anomalies to consider
PCH as defined by P. Barth [76–79]	PCH types 1 to 19 (neurodegenerative disorders with prenatal onset)
Cortical malformations [64–67, 99]	Lissencephaly (RELN, VLDRL, tubulin genes >> LIS1, DCX, ARX); polymicrogyria (tubulin genes, GPR56); periventricular nodular heterotopia (FLNA); primary microcephaly
Metabolic diseases [100]	Congenital disorders of glycosylation (mostly type 1a, but also in type 1q)
Genetic disorders [101, 102]	CASK mutation; cerebellar agenesis (PTF1 A)
Alpha-dystroglycanopathies [65, 68, 69]	Walker-Warburg syndrome; muscle-eye-brain disease; Fukuyama muscular dystrophy
Posterior fossa anomalies [81]	Pontine tegmental cap dysplasia
Disruptive lesions [102]	Cerebellar agenesis (most severe form; consider PTF1 A mutation)

*ARX*, aristaless related homeobox; *CASK*, calcium/calmodulin-dependent serine protein kinase; *DCX*, doublecortin; *FLNA*, protein filamin A; *GPR56*, adhesion G protein-coupled receptor G1; *LIS1*, lissencephaly 1; *PCH*, pontocerebellar hypoplasia; *PTF1 A*, pancreas transcription factor 1 subunit alpha; *RELN*, reelin; *VLDRL*, very low-density lipoprotein receptor

### Joubert syndrome and related disorders

**Fig. 13:**
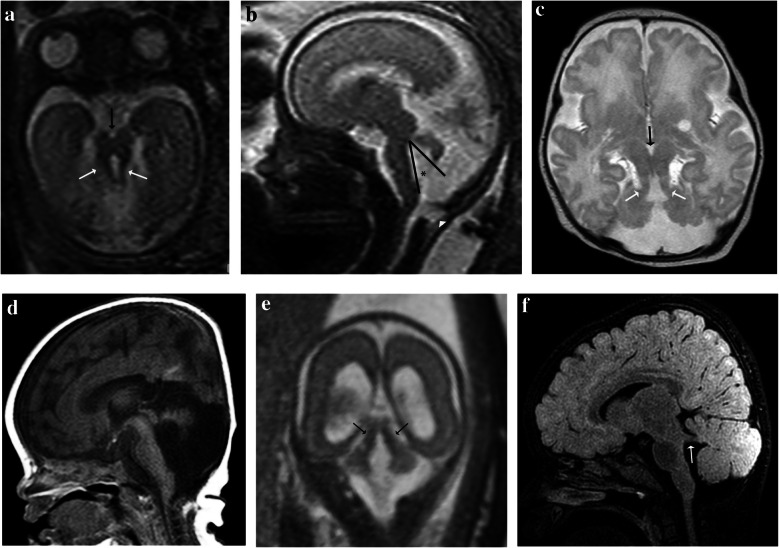
Joubert syndrome. Fetal MRI T2 SSFSE images at 23 weeks gestational age are notable for (**a**) the molar tooth sign in the axial plane, created by a deep interpeduncular cistern (*black arrow*) and thickened, horizontally oriented superior cerebellar peduncles (*white arrows*). The midline sagittal view (**b**) shows diffuse vermian hypoplasia (as opposed to an inferior-predominant vermian hypoplasia that is more typical of DWM) and a widened tegmento-vermian angle (*asterisk*), plus a suboccipital meningocele (*white arrowhead*). Postnatal brain MRI (**c**) axial T2 and (**d**) sagittal T1 redemonstrate these findings of Joubert syndrome. **e** A different fetal MRI T2 SSFSE coronal image at 22 weeks gestation shows a thickened appearance of the superior cerebellar peduncles (*black arrows*). **f** Postnatal brain MRI at 18 months of age demonstrates thickened and horizontally oriented superior cerebellar peduncles when viewed in a paramedian sagittal plane (*white arrow*)

Joubert syndrome (JS) is a rare inherited cerebellar ataxia that was first described by Joubert et al. in 1969 [82]. With subsequent advancements in neuroimaging, a common brainstem malformation was described as pathognomonic for these individuals - the molar tooth sign. Thickened and elongated superior cerebellar peduncles, a deep interpeduncular fossa, and a narrow pontomesencephalic junction resemble the shape of a molar tooth when viewed in the axial plane at the level of the midbrain (Fig. 13a). The recognition of other syndromes exhibiting the molar tooth sign has led to the expanded diagnostic term, Joubert syndrome and related disorders (JSRD) [83].

Joubert syndrome pathogenesis arises from impaired cilia function (ciliopathies), which affects the development of the brain, retina, kidneys, liver, and bones [84]. JS and Meckel-Gruber syndrome represent the mild and severe ends, respectively, of a spectrum caused by mutations in the same genes [85, 86]. More than 30 JSRD genes have been identified; however, these do not account for all patients [86]. Clinical features of JS in neonates include hypotonia, abnormal respiratory patterns, abnormal eye movements, and developmental delays. Some JS patients may exhibit only neurological abnormalities, although multisystem organ involvement can occur, manifesting with fibrocystic kidney and liver disease, retinal dystrophy, colobomas, occipital encephalocele, and polydactyly [86].

Prenatal diagnosis is challenging [87, 88] but important, both due to the typically poor prognosis and the risk of recurrence in subsequent pregnancies, given the recessive mode of inheritance in most cases. A high pre-test probability based on family history enhances prenatal detection of the molar tooth morphology. Scrutiny of brainstem morphology should be conducted in any case where cerebellar hypoplasia and/or vermian hypoplasia or absence of the vermis is present to evaluate for the molar tooth sign, although the presence of this sign varies across cases. An unusual shape of the fourth ventricle on axial and sagittal planes may provide a helpful clue: elongation in the AP direction, instead of the typically wider transverse dimension in the axial plane, along with blunting of the fastigial point and quadrangular morphology in the sagittal plane, has been described in these cases [89]. Additional findings suggesting the diagnosis include an enlarged posterior fossa, occipital cephalocele (Fig. 13), renal cystic disease, or polydactyly [75]. When posterior fossa enlargement occurs, these cases may be confused with Dandy-Walker malformations; therefore, scrutinizing the brainstem morphology for the molar tooth sign is important in cases of suspected DWM. Of note, the pattern of vermian hypoplasia may help in identifying JS. While DWM is characterized by inferior-predominant vermian hypoplasia, the vermian hypoplasia in JS is typically diffuse or anterior- (superior-) predominant (Figure 13).

### Kinked brainstem, L1CAM mutation, and diencephalon-mesencephalon junction dysplasia

**Fig. 14:**
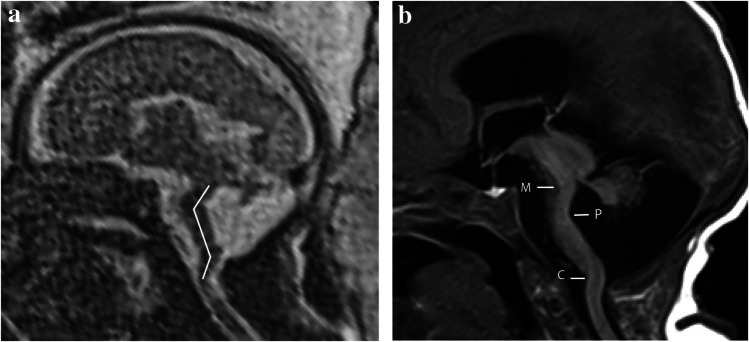
Examples of kinked brainstem. Fetal MRI (**a**) sagittal T2 SSFSE midline brain image at 26 2/7 weeks shows abnormal brainstem formation with kinked deformity and severe vermian hypoplasia. The suspected diagnosis was a tubulin mutation, although a genetic diagnosis was not made. Postnatal brain MRI of a different infant with Walker-Warburg (**b**) shows abnormal exaggeration of mesencephalic (M), pontine (P), and cervical (C) flexures of the brainstem on a sagittal T1-weighted image through the midline posterior fossa

As previously demonstrated, the dorsal aspect of a normal fetal brainstem is straight. An exaggerated flexure of the developing brainstem, appearing “kinked” or “zig-zag,” indicates severe dysgenesis [90]. This abnormal morphology reflects the shape of the primitive brainstem, featuring persistent bends at the mesencephalic, pontine, and cervical flexures (Fig. 14), which signify the arrested development of the brainstem [90]. Several genetic etiologies have been identified with this type of brainstem dysgenesis, including alpha-dystroglycanopathies, tubulinopathies, and L1 syndrome (X-linked congenital aqueductal stenosis and L1CAM mutations) [71, 90–92].

**Fig. 15:**
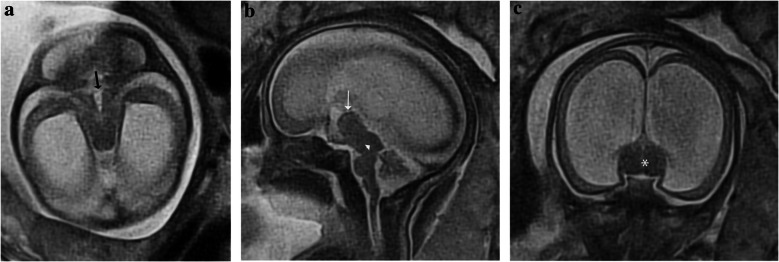
Diencephalic-mesencephalic junction dysplasia and L1CAM mutation variant of unknown significance. Fetal MRI was performed at 22 weeks for further evaluation of severe ventriculomegaly identified sonographically. Axial (**a**) T2 single-shot fast-spin echo (SSFSE) image through the brainstem shows abnormal contour with deep ventral cleft (*black arrow*). Sagittal (**b**) SSFSE image demonstrates an enlarged and ventrally positioned interthalamic adhesion (*white arrow*), nonvisualization of the cerebral aqueduct (*white arrowhead*), and normal size and shape of the fourth ventricle. Coronal (**c**) SSFSE image shows diencephalosynapsis (*asterisk*) and severe ventriculomegaly

L1CAM encodes a cell adhesion molecule, and pathogenic variants of L1CAM are known to be responsible for a spectrum of X-linked disorders, including X-linked hydrocephalus with aqueductal stenosis, developmental delay, aphasia, adducted thumbs, and corpus callosum dysgenesis [92]. Brain imaging findings include supratentorial obstructive hydrocephalus, corpus callosal dysgenesis, tectal plate enlargement, interthalamic adhesion hypertrophy, and vermian hypoplasia [93]. A fairly recent series of nine fetal cases with L1CAM pathogenic variants shared structural abnormalities, including callosal anomalies, reduced opercularization, diencephalosynapsis, brainstem kinking, and features of diencephalic–mesencephalic junction dysplasia (DMJD), a rare midbrain–hindbrain malformation [92]. DMJD is characterized on imaging by either abnormal cleavage between the midbrain and hypothalamus -- wherein a ventral midbrain cleft is continuous with the third ventricle, creating a “butterfly-like” appearance of the brainstem in the axial plane -- or by union between the midbrain and thalamus in the sagittal plane [94–96] (Fig. 15). DMJD is variably associated with other brain malformations, including corpus callosal dysgenesis and obstructive ventriculomegaly. DMJD has also been described in association with tubulinopathies. Affected individuals may exhibit severe cognitive impairment, progressive microcephaly, hypotonia, spastic quadriparesis, and seizures [94, 95]. Of note, reports of DMJD caused by L1CAM mutations do not signify that all cases of L1 syndrome include this dysplasia. Nevertheless, careful inspection of the fetal brainstem on both ultrasound and MRI for morphology and clefts is important in all cases, particularly so in the setting of supratentorial ventriculomegaly.

## Conclusion

Infratentorial anomalies are associated with a wide range of etiologies. With recent advances in genetic diagnostics, our understanding of the molecular mechanisms behind these disorders continues to grow. Observing anatomy through prenatal imaging can facilitate the categorization of posterior fossa cystic anomalies, whether with or without posterior fossa enlargement, primary cerebellar dysgenesis, or brainstem involvement. This process narrows the diagnostic considerations for a more focused prenatal assessment and enhances counseling efforts.

## Data Availability

No datasets were generated or analysed during the current study.
